# Radiotherapy-Compatible Robotic System for Multi-Landmark Positioning in Head and Neck Cancer Treatments

**DOI:** 10.1038/s41598-019-50797-7

**Published:** 2019-10-07

**Authors:** Mark Ostyn, Siqiu Wang, Yun-Soung Kim, Siyong Kim, Woon-Hong Yeo

**Affiliations:** 1 Department of Radiation Oncology, Medical Physics Graduate Program, School of Medicine, Virginia Commonwealth University, Richmond, VA 23298 USA; 2 George W. Woodruff School of Mechanical Engineering, Institute for Electronics and Nanotechnology, Georgia Institute of Technology, Atlanta, GA 30332 USA; 3 Wallace H. Coulter Department of Biomedical Engineering, Parker H. Petit Institute for Bioengineering and Biosciences, Institute for Materials, Georgia Institute of Technology, Atlanta, GA 30332 USA

**Keywords:** Engineering, Techniques and instrumentation

## Abstract

The spine flexibility creates one of the most significant challenges to proper positioning in radiation therapy of head and neck cancers. Even though existing immobilization techniques can reduce the positioning uncertainty, residual errors (2–3 mm along the cervical spine) cannot be mitigated by single translation-based approaches. Here, we introduce a fully radiotherapy-compatible electro-mechanical robotic system, capable of positioning a patient’s head with submillimeter accuracy in clinically acceptable spatial constraints. Key mechanical components, designed by finite element analysis, are fabricated with 3D printing and a cyclic loading test of the printed materials captures a great mechanical robustness. Measured attenuation of most printed components is lower than analytic estimations and radiographic imaging shows no visible artifacts, implying full radio-compatibility. The new system evaluates the positioning accuracy with an anthropomorphic skeletal phantom and optical tracking system, which shows a minimal residual error (0.7 ± 0.3 mm). This device also offers an accurate assessment of the post correction error of aligning individual regions when the head and body are individually positioned. Collectively, the radiotherapy-compatible robotic system enables multi-landmark setup to align the head and body independently and accurately for radiation treatment, which will significantly reduce the need for large margins in the lower neck.

## Introduction

The fundamental challenge in achieving accurate positioning in head and neck radiotherapy is that the anatomy at and above the cervical spine does not act as a single rigid body. Instead, the anatomy is comprised of individually articulated structures between the thorax and skull that are mechanically flexible. This flexibility creates significant uncertainties in the reproducibility of a patient-specific setup position. Therefore, in addition to irradiating the primary treatment site, additional margins of healthy tissue around the tumor are irradiated to ensure coverage of the target in the event of an unintended setup error. The size of these margins are proportional to the degree of uncertainty known to be present in typical clinical setup positioning^1,2^ While the use of margins increases the probability of adequate target coverage, irradiating healthy tissue increases the risk of harmful side effects to the patient, such as xerostomia or secondary cancer^3,4^. To reduce the uncertainty caused by the neck flexibility, passive immobilization masks are employed to reduce motion of the patient and to recreate setup positioning between different days of treatment. However, previous studies^1,5–10^ have demonstrated that these rigid shoulder-to-face-covering masks do not fully mitigate this uncertainty with typical setup errors of 3–5 mm between distant landmarks along the spine of the neck. Recent reports^11–15^ show that an accurate positioning in head and neck therapy can be achieved by independent positioning of the head from the rest of the body. However, these mechanical systems have not yet been introduced into regular clinical practice. While these works have presented electromechanical systems, none of these systems are fully radiotherapy-compatible. The placement of high-atomic number (Z) materials in the irradiated region can excessively interfere with X-ray setup guidance and therapeutic X-ray delivery. Even though our prior work^11^ uses primarily low-Z plastics, this system still includes metallic motors near the treatment region or clinically infeasible locations, which limits the clearance of the linear accelerator gantry.

Here, we introduce a fully radiotherapy-compatible robotic system for multi-landmark positioning in head and neck radiotherapy. In this new system, all radiotherapy-incompatible components are arranged far outside of the radiation field, placed 40 cm inferior to radiation isocenter with a novel, plastic gearbox that reduces linear accelerator gantry clearance issues. As a result of transitioning from direct to indirect power transmission from stepper motors to end effectors, a mostly-plastic secondary position feedback is introduced to aid in navigation. We validate the system’s radiotherapy compatibility via attenuation measurement and radiographic imaging. Demonstration of the positioning accuracy with an anthropomorphic skeletal phantom captures the clinical applicability of the electromechanical robotic system. To our knowledge, this is the first study to examine the residual errors in the neck when independently positioning the head from the rest of the body.

## Results and Discussion

### Design and fabrication of a robotic system

**Figure 1:**
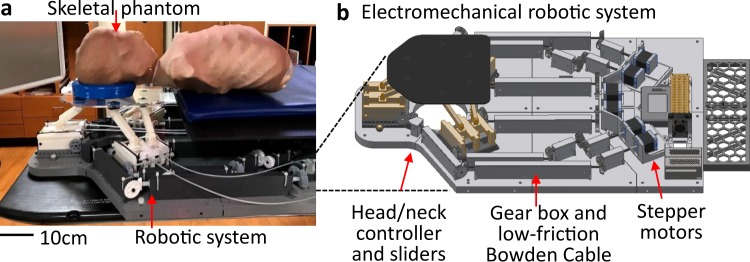
Fully radiotherapy-compatible robotic system. (**a**) Photo of a skeletal phantom on the robotic system, which is added on the existing treatment couch for head and neck cancer. (**b**) Schematic illustration of the robotic system, including head/neck plastic controller, plastic gearbox and low-friction wires, and stepper motors located far outside the radiation field.

One of the main challenges in the design of a fully radiotherapy-compatible robotic system is to make it as compact as possible, while still maintaining a meaningful range of control motions. Previous systems^12–14^ that aim to adjust the head and neck positions have taken the form of an exertion of the couch top. The idea is to put all metal mechanical components placed below the patient’s head or as a separate accessory placed between the couch top and patient head. Any material near the regions of interest affects radiographic imaging and delivered dose of radiation. In this work, to minimize this interference, we place a significant portion of key mechanical components (metal motors) in-between the patient’s body and the couch top. Figure 1 captures an example of a prototype device that we designed; a mechanical robotic system is added on the existing treatment couch for head and neck cancer. This system was designed to specifically control the position of a patient’s head separately from the remainder of their body (Fig. 1a), while majority of the mechanical components are located under their body (Fig. 1b). For radiation compatibility, 3D printing was chosen to fabricate nearly all major components, including the gear box, end effector assembly, enclosure, and mounting frame. Two types of printers can manufacture those components. For high resolution printing that requires features smaller than 1 mm, we use Formlabs Form 2 printer and Objet Eden 260VS. On the other hand, components that do require high detail can be printed with a combination of fused deposition modeling printers, including uPrint SE Prototyper, MakerBot Z18, and MakerBot Replicator. The 3D printable materials selected for the robotic system include acrylonitrile butadiene styrene (ABS) when using the uPrint printer, polylactic acid (PLA) for any MakerBot printer, standard resin for the Formlabs, and polymer (VeroWhitePlus RGD835) for the Objet Eden. In addition, the top plate of the head/neck controller (Fig. 1b) that needs an extra high strength is fabricated by machining a carbon-fiber-reinforced plastic, which holds a patient’s head.

**Figure 2:**
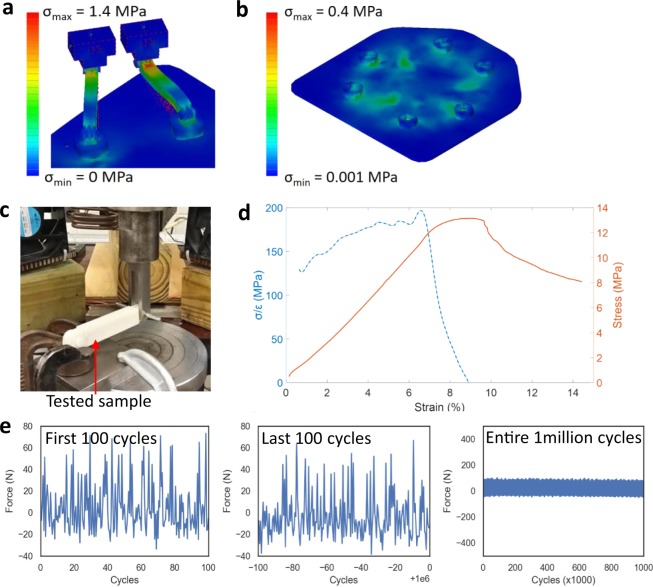
Mechanical analysis of the head and neck controller. (**a,b**) FEA results of the estimated maximum stress occurred on the linkage (**a**) and top positioning plate (**b**). (**c**) Photo of a cyclic fatigue tester of a 3D printed linkage. (**d**) Measured stress-strain curve of an ABS plastic-based linkage (orange: original stress-strain curve and blue: calculated ratio of stress and strain). (**e**) Result of the fatigue test up to 1 million cycles, showing negligible change of values over cycles.

To design a mechanically stable system for clinical applications, we used a computational mechanics modeling based finite element analysis (FEA)^16^. In this analysis, a simplified geometry of the linkages, hinges, sliders, and positioning plate was used where the entire assembly was simulated as ABS plastic. A normal force of 75 N that estimates the weight of a human head is directly applied to the linkages and head positioning top-plate (Fig. 2a,b). With an elliptical load distribution, FEA based on Autodesk Inventor estimates the von Mises stress to determine if a given material is yielded or fractured. The estimated maximum stress on the structure linkage and plate is only 1.4 and 0.4 MPa, respectively (Fig. 2a,b). Comparison with the allowable yield strength (40 MPa) of the material (ABS plastic) supports the mechanical safety of the designed structure. In addition, a dynamically loaded force (75 N) was applied to the positioning plate with a 1-second duration. The result indicates that the linkages are safe up to ~10^9^ cycles, which would be a reasonable lifetime of the system.

To verify the FEA study, a printed linkage was experimentally analyzed to measure empirical elongation at breaking, modulus of elasticity, and fatigue properties (Fig. 2c–e). To test the elongation at break and the modulus of elasticity, a sample linkage was gripped in a universal testing machine (Fig. 2c) and force was applied. The linkage was gripped at each end on the flat edge of each hinge. The estimated applied stress was the applied force divided by the cross-sectional area of the gripped segment of the linkage. The physical stress analysis (stress-strain curve in Fig. 2d) shows that the 3D-printed linkage is substantially weaker than a bulk ABS plastic. The calculated value of Young’s modulus for the printed component is ranged from 130 to 200 MPa, while the typical ABS plastic has ~9 GPa^17^. Additionally, the ultimate elongation is about 9%, which is approximately in line with values for ABS plastic from a prior report^18^. Although the measured mechanical strength for the 3D printed material was substantially lower than the bulk, the resultant part can be still rated as strong enough to support a patient’s head. The fracture tensile loading of the linkage is 1180 N, compared to the expected upper limit of 75 N, distributed between 6 linkages of the head and neck system.

A fatigue test of a sample joint (Fig. 2e) consisting of a linkage, hinge, and connecting pin shows no mechanical fracture with 1 million cycles, which indicates that the plastic components are durable enough to support the expected patient load *in vivo*. Although the applied forces in the fatigue analysis were less than the weight of a typical human head, the load is distributed among six linkages in the full assembly, such that the typical loading will be close to 12.5 N. Through our measurements, the linkage tolerated approximately 730 × 10^3^ loadings with compressive force greater than this value, approximately 150 × 10^3^ loadings with compressive force greater than 50 N and 1.4 × 10^3^ loadings with compressive force greater than 75 N without breaking. We anticipate that a typical workload for this system would involve up to 10 patients per weekday, for approximately 2,000 loadings per year.

### Mechanism of a position feedback loop

**Figure 3:**
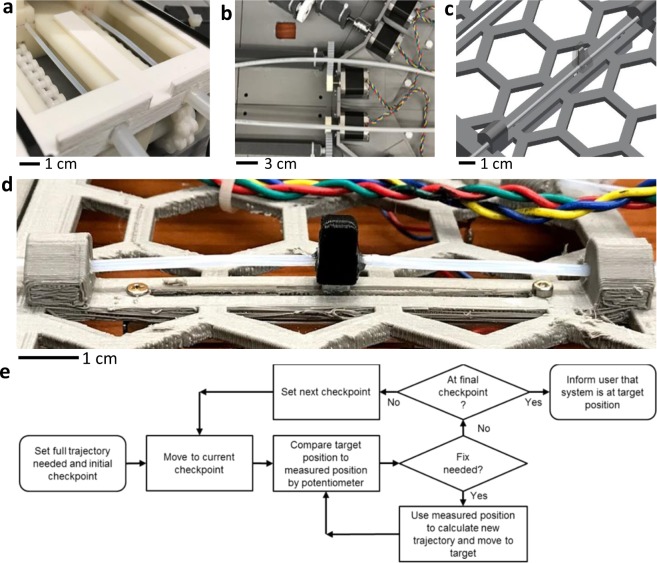
Voltage measurement-guided feedback loop system. (**a**) Zoomed-in view of fixed tubes and low-friction wires. (**b**) Paths of wires and tubes along with stepper motors. (**c**) Illustration of a wire passing through a linear potentiometer lever. (**d**) Photo of the potentiometer in the middle. (**e**) Flow chart of the feedback loop system.

For the robotic system, lashing, occurred in the gearbox, prohibits dead reckoning for slider navigation. Therefore, a voltage-guided feedback loop devises as a physical measurement of slider positions (Fig. 3). In this scheme, the sliders (Fig. 3a–d) follow prescribed motion trajectories broken down into checkpoints every *n* steps. At each checkpoint, the position of each slider is measured by using a voltage-based surrogate and the remaining trajectory is updated based on the most recent localization information. A series of flexible Bowden cables was devised to create a mechanical connection between each slider and a corresponding sliding potentiometer (linear slide potentiometer, Bourns) located far outside the treatment field. The cable assemblies were created by using nylon tubing and 2-mm diameter wires (polytetrafluoroethylene; PTFE). The synthetic polymer (PTFE) was selected due to its low coefficient of friction to minimize drag against the tubing. The inner diameter of the tubing was 19% larger than the diameter of the wire (difference: 380 μm). These dimensions were chosen as the best compromise between off-the-shelf discrete sizes, component thinness for flexibility, and thickness for durability. A data acquisition system (National Instruments) was used to power and read the potentiometer data. Because the measured voltages on the potentiometer were very noisy when the stepper motors were powered, a 100-point moving average was used, which introduced a 1-second latency period. A flow chart in Fig. 3e describes the overview of the feedback loop, starting from the initial checkpoint to the final target position.

### Mechanism for an absolute positioning accuracy

**Figure 4:**
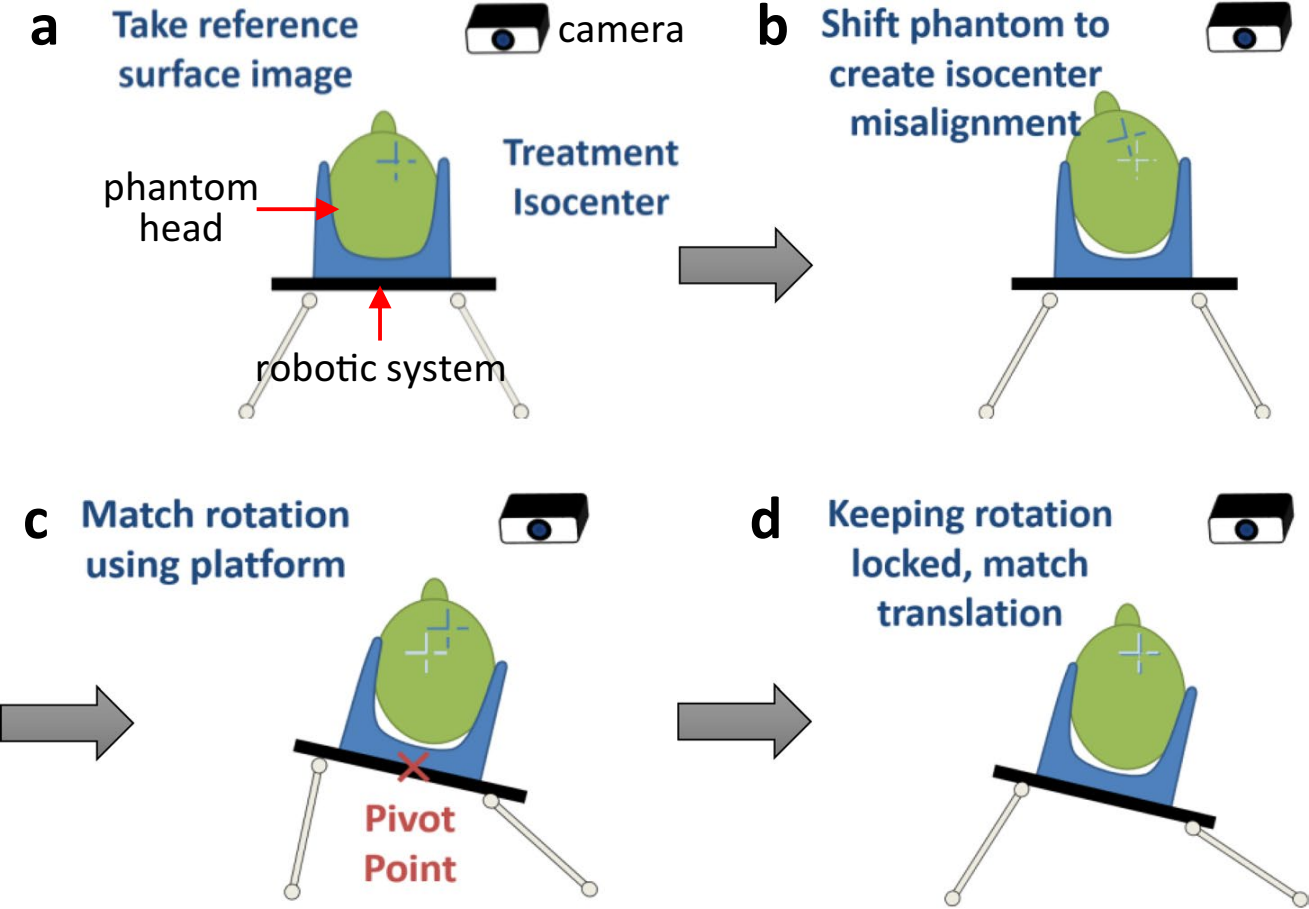
Mechanism for an absolute positioning accuracy. (**a–d**) Sequential workflow to achieve an absolute accuracy of a patient positioning on the radiation treatment table.

Figure 4 describes a sequential workflow to achieve an absolute accuracy of a patient positioning on the radiation treatment table. We used an optical surface monitoring system (AlignRT) to evaluate the position of a skeletal phantom used in this study, which determined the performance of the feedback loop system. This phantom was used as a patient surrogate for correction. Since there was friction between the smooth nylon mesh of the phantom and a headrest typically used in clinic, we utilized a rubberized grip ring instead of the headrest in this simulation. This arrangement was helpful to control the load distribution of the skull to ensure that load concentration remained within the points of stability on the plate.

The evaluation test consists of 15 independent correction trials where the head was set up at an arbitrary position on the grip, then marked as a reference position (Fig. 4a). An arbitrary misalignment is then created (Fig. 4b) by manual manipulation. Negations of the detected displacements were used as correction vectors, first applying a correction for rotations, followed by the resultant measured translation displacements. This process resolves the problem of alias translations, created by discrepancies between pivot points used in the surface tracking system and in the plate positioning software (Fig. 4c). The software allows a customizable reference pivot point for rotation motions (Fig. 4d). In an effort to reduce the magnitude of alias translations, the pivot point is placed approximately at the location of the C2 vertebrae (90 mm inferior to the center of the plate, 60 mm above the anterior surface of the plate), which is based on the observation that the rigid registrations of C2 provided the best alignments of bony landmarks^8^.

### Quantification of radiographic compatibility of the robotic system

All materials used in the positioning robotic system were carefully selected to minimize the effects of scatter and attenuation during radiotherapy. Based on the similar method from our prior work^11^, we conducted a more rigorous method for prospectively assessing radio-compatibility for any arbitrary material. All components within 40 cm horizontally of the positioning system’s isocenter were constructed by fully using low-Z materials. These materials were selected based on the estimated attenuation, as listed in Table 1, which was generated by using the basic definition of Hounsfield Units (HU)^19^ and comparing the estimated HU values to the reported value of a compact bone tissue (2500 HU).

**Table 1:** Estimated attenuation of materials used in the robotic system.

Material	Chemical composition	*ρ* (g/cm^3^)	*μ/ρ* (40 keV)	Estimated attenuation (HU value)
PLA	C_3_H_4_O_2_	1.25	0.214	0
Tough PLA	C_2_H_4_O_2_	1.5	0.214	200
ABS	C_15_H_17_N	1.05	0.220	−140
Nylon	C_12_O_2_N_2_H_22_	1.13	0.231	−30
CFRP	67% C; 33% C_21_H_25_ClO_5_	1.44	0.241	300
POM	CH_2_O	1.41	0.244	290
PMMA	C_5_H_8_O_2_	1.19	0.235	40
PTFE	C_2_F_4_	2.20	0.265	1180
Water	H_2_O	1.00	0.268	0
Air	78% N; 21% O; 1% Ar	1.29 × 10^−3^	0.246	−1000
*Bone* *(reference)*	—	—	—	2500

When a material’s HU value is smaller than that of the bone tissue, then it is deemed likely compatible with radiographic imaging. The HU value is calculated by the following equation:

1 $$H{U}_{material}(E)=\frac{{\mu }_{material}(E)-{\mu }_{water}(E)}{{\mu }_{water}(E)-{\mu }_{air}(E)}$$

where *μ* is the linear attenuation coefficient of the material in question, and that for water and air and *E* is the energy of the incident x-rays. The net linear attenuation coefficient for each material is calculated by treating each material like a mixture^20^:

2 $${(\frac{\mu }{\rho })}_{mixture}(E)\cong {(\frac{\mu }{\rho })}_{A}(E){f}_{A}+{(\frac{\mu }{\rho })}_{B}(E){f}_{B}+\ldots $$

where *ρ* is the density of each material and *f* is the fractional weight of each material in the mixture. The composition of most used materials is well known, but some materials used are less established. The carbon fiber-reinforced polymer (CFRP) is a mixture of carbon fibers and a reinforcing epoxy resin. While the exact ratios of carbon fiber to epoxy are unknown for the materials used, the mix was estimated to be 2:1 of carbon:epoxy by mass. The chemical composition for carbon fibers is simply pure carbon, but many possible forms of epoxy could have been utilized in the formation of the materials used in this work. Since the purpose of this exercise is to obtain an estimated attenuation, one possible chemical composition for epoxy is (C_21_H_25_ClO_5_)_n_. Table 1 summarizes all of nominal chemical compositions and densities of the materials used to build the robotic system. Overall, all of the materials used in the system show smaller HU values compared to the reference (bone tissue).

**Figure 5:**
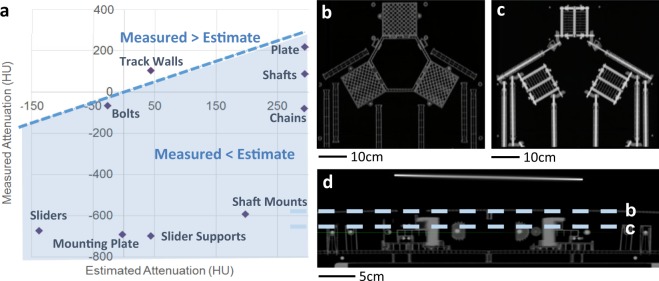
Validation of radio-compatibility. (**a**) Comparison between estimated attenuation and measured attenuation of materials used in the system. (**b**) Horizontal CT slice through the base of the system. (**c**) Horizontal slice through the vertical height of the axles and gears. (**d**) Side-view of radiographic image, dashed lines indicate vertical heights of slices shown in (**b,c**). These sectional CT images show no visible artifacts.

To validate the estimation, the fabricated device was placed in a CT simulation unit for actual measurement of HU values (Fig. 5a). As can be seen in Fig. 5a, measured values of attenuation are smaller than the analytic estimates in most cases, which is good in radio-compatibility aspect. In many cases, non-load bearing components were designed to utilize a minimal amount of plastic to reduce production costs and production time. These components were often printed as plastic shells with reinforcing ribs using thicknesses 2 or 3-mm shells and 1-mm thickness ribs. Due to this design practice, some components lacked volumetric regions spatially larger than the voxel size of the CT scanner, resulting in significant volume between the air and the plastic components. In addition, we conducted radiographic imaging of the fabricated system (Fig. 5b–d), which clearly shows no visible artifacts. These images that capture sectional CT reconstruction of top views and side view validate the system’s full radio-compatibility.

It should be noted that the focus of this work was on the demonstration of a framework for estimating the radiotherapy-compatibility of materials in use through the estimation of CT HU values. Though we have only demonstrated the compatibility for some common synthetic polymer materials, many other plastic materials share similar atomic composition and density, indicating a wide variety of options materials suitable for construction of a final product. Although this system was constructed from largely 3D-printed materials, the final prototype for a clinical study will utilize higher quality materials along with more robust construction methods.

### Analysis of absolute positioning error and independent head correction

**Figure 6:**
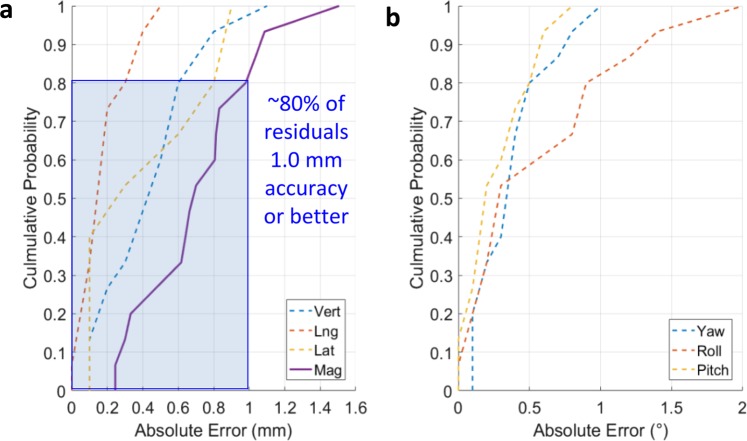
Analysis of absolute positioning error. (**a**) Measured absolute translation error (mm) showing the accuracy of 0.7 ± 0.3 mm. (**b**) Absolute rotation errors in yaw, roll, and pitch, showing the accuracy of all below 3°.

Figure 6 summarizes the absolute positioning errors in both translation and rotation via cumulative histograms. With 15 trials, the mean absolute displacements for correction ranges from 1.1 to 5.2 mm (distribution of 2.5 ± 1.2 mm). The angular displacements were up to rotations are all below 3° to ensure that the position would always be achievable. The mean absolute vector displacement for post correction is 0.7 ± 0.3 mm, indicating the robotic system’s submillimeter accuracy. As indicated in Fig. 6a, approximately 80% of error residuals are of 1 mm magnitude or less. This corresponds to the normal distribution of 0.7 ± 0.3 mm would be less than 1 mm, counting all data below the mean values plus one standard deviation.

The mechanical positioning accuracy of this system was less than that of other similar robotic applications, such as those presented by Liu^12^ or Ostyn^11^. For example, our previously introduced system had a positioning error of 0.4 ± 0.3 mm error, compared to the 0.7 ± 0.3 mm of the system in this work. While this is less desirable, this system contains significantly greater constraints on construction that other similar prototypes, especially in the requirements for non-metal construction for the vast majority of the device so that the device is usable in a clinical setting. This is the most significant trade-off within this work: in the first-of-its-kind mostly plastic construction, mechanical accuracy was reduced, but the system would not excessively interfere with X-ray-based imaging or treatment delivery. However, even with this lower accuracy, the positioning accuracy of the presented system stands to significantly reduce typical errors (3–5 mm) seen in head and neck radiotherapy and could be used to justify reducing the amount of healthy tissue being irradiated.

**Figure 7:**
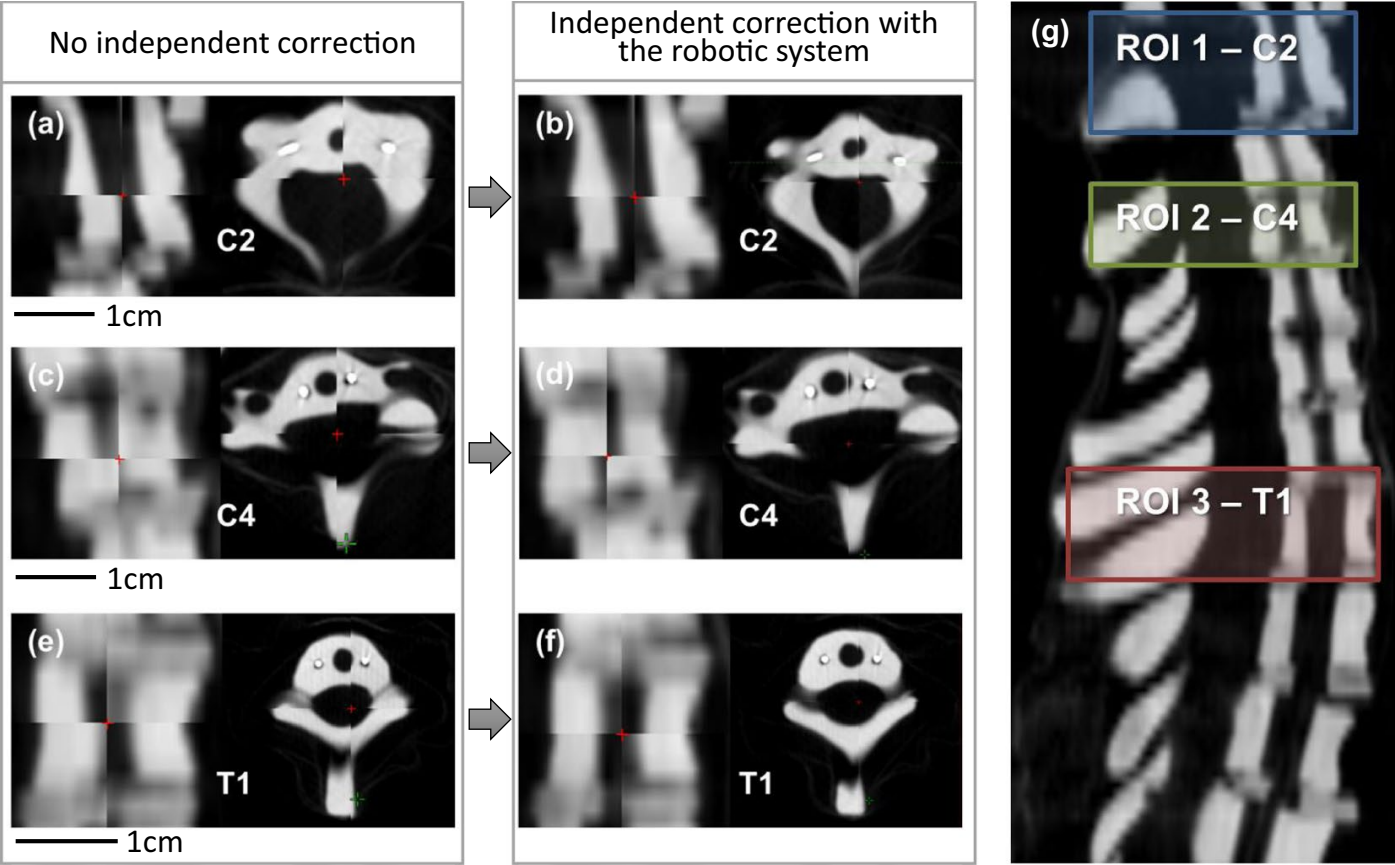
Comparison of no independent correction of CBCT images with the case of the robotic system-assisted correction. (**a,c,e**) CBCT images without independent correction for C2, C4, and T1, respectively. (**b,d,f**) CBCT images that are independently corrected by the robotic system for C2, C4, and T1, respectively. (**g**) Regions of interest of C2, C4, and T1 in a skeleton figure.

To validate the system’s performance, we examined the impact of independent head positioning on sub-regions of interest along the thorax (Fig. 7). This study followed the same basic method, used to measure positioning accuracy with a few steps added. Before any mechanical correction was attempted, the entire length of the anthropomorphic skeleton was manually set up to be as close as possible to the planning CT image. Once in alignment, a reference CT image was taken to be used as the source for registration of misaligned and corrected images. Afterwards, a series of 3 trials were performed to correct manually created positioning errors where the rotations were corrected first, followed by correcting the translations. After the manual misalignment and each correction step, cone-beam CT (CBCT) images were taken, which were then rigidly registered to the reference image at three separate regions of interest, including C2, C4, and T1. To reduce confusion about alias transformations (rotation-induced translations), registrations of translations and rotations were performed separately. Table 2 summarizes residual errors before and after independent correction by the developed robotic system. For translation vector error, the post correction by the robotic system decreases the residual errors from C2, C4, and T1, compared to the simulated setup error. As expected, the total rotation errors (pitch + roll + yaw) are also decreased by the post correction.

**Table 2:** Residual errors before and after independent correction by the robotic system.

	Anatomical region	Translation error (mm)	Rotation error (pitch + roll + yaw) (°)
Simulated setup error	C2	3.4 ± 2.4	2.7 ± 1.2
	C4	2.5 ± 2.1	1.6 ± 0.8
	T1	1.0 ± 0.8	1.1 ± 0.9
Post correction error	C2	1.3 ± 0.7	1.2 ± 0.3
	C4	1.0 ± 0.4	0.5 ± 0.3
	T1	0.8 ± 0.2	0.5 ± 0.3

To our knowledge, this is the first study that has examined the impact of independent head positioning on sub-regions of interest along the neck by an electromechanical system. The presented set of results indicate that the uncertainty in positioning sub-regions of the neck can be significantly reduced as a result of careful positioning of individual landmarks on either end of the neck compared to rigid translations of the greater anatomical region.

## Conclusions

We have introduced the first demonstration of a fully radiotherapy-compatible electro-mechanical robotic system for head and neck cancer therapy. This presented system is capable of positioning a patient’s head with submillimeter accuracy in clinically acceptable spatial constraints. Calculated attenuation of materials in the system have lower HU values than a reference material (bone tissue), which is validated by a set of radiographic images with no visible artifacts. The result of positioning accuracy with a skeletal phantom determines the device’s accuracy as 0.7 ± 0.3 mm. In addition, CBCT imaging and post correction study validates the system’s functionality for aligning individual regions when the head and body are individually positioned. Future work will focus on the radiotherapy application for patients with head and neck cancer.

## Methods

### Mechanical cyclic fatigue test

Two 3D printed ABS parts comprising a single joint of a linkage was assembled to experimentally monitor the fatigue-related damages from cyclic actuation. A uniaxial servohydraulic testing machine (MTS Systems, Inc) was used to vertically displace the joint at 5 Hz with a fixed displacement of 4.8 mm up to 1 million cycles while recording the vertically exerted forces. Computer software was programmed to monitor changes of 5% or greater in the recorded peak forces and automatically complete the test.

### Geometric optimized algorithm

A constraints-based geometric optimization algorithm was applied to determine feasible dimensions for the end effector assembly that would maximize the 6D range of motion while remaining within set constraints of dimensional size in the lateral and vertical dimensions. The optimizer scanned through a set of permutations of the relevant variable dimensional parameters of the end effector assembly testing to see whether an assembly constructed to the candidate dimensions could mechanically achieve a set of configurations at the limits of a set range of motion. This optimization process produced a set of dimensions for the prototypical end effector assembly that minimally extended off the sides of the patient support assembly couch top, was less than 17 cm in height in rest position and could mechanically achieve any position within a range of ±8 mm translationally and simultaneously ±3° rotationally.

## References

[1] Cheo T, Loh Y, Chen D, Lee KM, Tham I (2015). Measuring radiotherapy setup errors at multiple neck levels in nasopharyngeal cancer (NPC): A case for differential PTV expansion. Radiother Oncol.

[2] van Herk M, Remeijer P, Rasch C, Lebesque JV (2000). The probability of correct target dosage: dose-population histograms for deriving treatment margins in radiotherapy. Int J Radiat Oncol Biol Phys.

[3] Bressan V (2016). The effects of swallowing disorders, dysgeusia, oral mucositis and xerostomia on nutritional status, oral intake and weight loss in head and neck cancer patients: A systematic review. Cancer Treat Rev.

[4] Hall, E. G., Amato. *Radiobiology for the Radiologist*. Seventh Edition edn, (Lippincott Williams and Wilkins, 2012).

[5] Zhang L (2006). Multiple regions-of-interest analysis of setup uncertainties for head-and-neck cancer radiotherapy. Int J Radiat Oncol Biol Phys.

[6] Polat B, Wilbert J, Baier K, Flentje M, Guckenberger M (2007). Nonrigid patient setup errors in the head-and-neck region. Strahlenther Onkol.

[7] Ove R, Cavalieri R, Noble D, Russo SM (2012). Variation of neck position with image-guided radiotherapy for head and neck cancer. Am J Clin Oncol.

[8] Graff P (2013). The residual setup errors of different IGRT alignment procedures for head and neck IMRT and the resulting dosimetric impact. Int J Radiat Oncol Biol Phys.

[9] Djordjevic M, Sjoholm E, Tullgren O, Sorcini B (2014). Assessment of residual setup errors for anatomical sub-structures in image-guided head-and-neck cancer radiotherapy. Acta Oncol.

[10] Piotrowski T (2013). Impact of the spinal cord position uncertainty on the dose received during head and neck helical tomotherapy. J Med Imaging Radiat Oncol.

[11] Ostyn M (2017). An electromechanical, patient positioning system for head and neck radiotherapy. Phys Med Biol.

[12] Liu X, Belcher AH, Grelewicz Z, Wiersma RD (2015). Robotic real-time translational and rotational head motion correction during frameless stereotactic radiosurgery. Med Phys.

[13] Wiersma RD, Wen Z, Sadinski M, Kang H (2010). Development of Frameless SRS using Real-Time 6D Facial Surface Monitoring and Continuous Adaptive Head Motion Correction. Int J Radiat Oncol.

[14] Wiersma, R., Belcher, A. & Grelewicz, Z. Development of a 4DOF Robotic Frameless SRS System for Both Translational and Rotational Head Motion Cancellation. *Medical Physics* **40**, 10.1118/1.4815688 (2013).

[15] Ostyn M, Kim S, Yeo W-H (2016). A simulation study of a radiofrequency localization system for tracking patient motion in radiotherapy. Sensors.

[16] Herbert R, Kim J-H, Kim Y, Lee H, Yeo W-H (2018). Soft material-enabled, flexible hybrid electronics for medicine, healthcare, and human-machine interfaces. Materials.

[17] Golub, M., Guo, X., Jung, M. & Zhang, J. In *REWAS 2016* 281–285 (Springer, 2016).

[18] Hassan MM (2008). Mechanical, thermal, and morphological behavior of the polyamide 6/acrylonitrile-butadiene-styrene blends irradiated with gamma rays. Polym Eng Sci.

[19] Bushberg, J. S., Anthony, J., Leidholt, E. & Boone, J. *The Essential Physics of Medical Imaging*. Third Edition edn, (Lippincott Williams and Wilkins, 2012).

[20] Attix, F. *Introduction to Radiological Physics and Radiation Dosimetry*. (John Wiley and Sons, Inc, 1986).

